# Prior medial meniscus arthroscopy is not associated with worst functional outcomes in patients undergoing primary total knee arthroplasty: A retrospective single-center study with a minimum follow-up of 5 years

**DOI:** 10.1051/sicotj/2024001

**Published:** 2024-01-18

**Authors:** Vasileios Giovanoulis, Axel Schmidt, Angelo V. Vasiliadis, Christos Koutserimpas, Cécile Batailler, Sébastien Lustig, Elvire Servien

**Affiliations:** 1 Orthopaedic Surgery and Sports Medicine Department, Croix-Rousse Hospital, University Hospital 69004 Lyon France; 2 Univ Lyon, Claude Bernard Lyon 1 University, IFSTTAR, LBMC UMR_T9406 69622 Lyon France; 3 Department of Anatomy, School of Medicine, Faculty of Health Sciences, National and Kapodistrian University of Athens 11527 Athens Greece; 4 LIBM – EA 7424, Interuniversity Laboratory of Biology of Mobility, Claude Bernard Lyon 1 University 69622 Lyon France

**Keywords:** Total knee arthroplasty, Knee arthroscopy, Knee anatomy, Arthroscopy outcomes, Knee surgery

## Abstract

*Introduction*: There have been controversial studies on the impact of prior knee arthroscopy (KA) on outcomes of total knee arthroplasty (TKA). The purpose of this comparative study is to investigate the impact of prior KA of medial meniscus on patients undergoing TKA by evaluating the International Knee Society Score (IKS), the complications, and revisions. *Methods*: This retrospective study reviewed 84 patients with TKA who had undergone prior KA of the medial meniscus and compared them to 84 cases, without a history of prior KA as a control group. Outcomes were assessed with the original IKS scores and complications. The mean follow-up was 8 years. *Results*: There was no significant difference between groups with respect to demographics, or pre-operative IKS. The mean pre and postoperative IKS was not different between groups. The all-cause reoperation, revision, and complication rates of the KA group were not significantly higher than those of the control group. *Conclusion*: The present study seems to reveal that previous KA of the medial meniscus does not negatively affect a subsequent TKA. Nevertheless, larger studies may be necessary to confirm this observation.

## Introduction

Total knee arthroplasty (TKA) is rising due to the increase in the lifetime expectancy of patients suffering from severe osteoarthritis (OA) which could be attributed to a more active lifestyle [1]. TKA is among the safest and most cost-effective surgeries in orthopedics. It aims to relieve the pain and improve the functional restoration and the quality of life for patients with advanced stages of knee OA [1]. Over time, joint reconstruction surgery has advanced and improved through the adoption of various techniques and technologies, including less invasive operative procedures, fast recovery protocols, better pre-intra and-post-operative management to reduce the need for blood transfusions, as well as outstanding improvements in navigation or robotic systems [2–5]. Additionally, modern and more “friendly” materials have been developed for prosthetic joints [2, 3, 6–8]. It has been reported that previous knee procedures, such as high tibial osteotomies and osteosynthesis of the tibial plateau or distal femoral fractures may result in inferior post-operative outcomes after performing a TKA [9]. Furthermore, knee arthroscopy (KA) may be frequently performed in patients with early OA to improve symptomology, but any possible short-term benefit usually does not persist after one year [10].

There is scarce data regarding the outcomes of primary TKA in patients who have undergone KA of medial meniscus pathology such as partial, subtotal, or total medial meniscectomy and/or debridement and meniscal repair. Some studies suggest that KA prior to TKA may increase complication rates [11]; however, others do not reveal a negative impact on TKA outcomes [12].

The purpose of the present study is to compare the outcomes of patients undergoing TKA after KA for pathologies of the medial meniscus (study group) to patients undergoing TKA without prior KA (control group), by evaluating the International Knee Society Score (IKS), the complications and revisions.

## Material and methods

The present is a retrospective comparative study of a prospectively maintained database. A total of 905 patients who underwent primary TKA from January 2007 to April 2016 at the Orthopaedic Surgery and Sports Medicine Department, Croix-Rousse Hospital, University Hospital, Lyon, France were screened. All patients had a posterior stabilized type implant system (Corin – HLS KneeTec^™ ®^) according to the surgeon’s preference and the implant choice commonly used in this department. Surgery was performed through a medial-midline longitudinal skin incision and a medial parapatellar or sub-vastus arthrotomy.

## Patients

**Figure 1:**
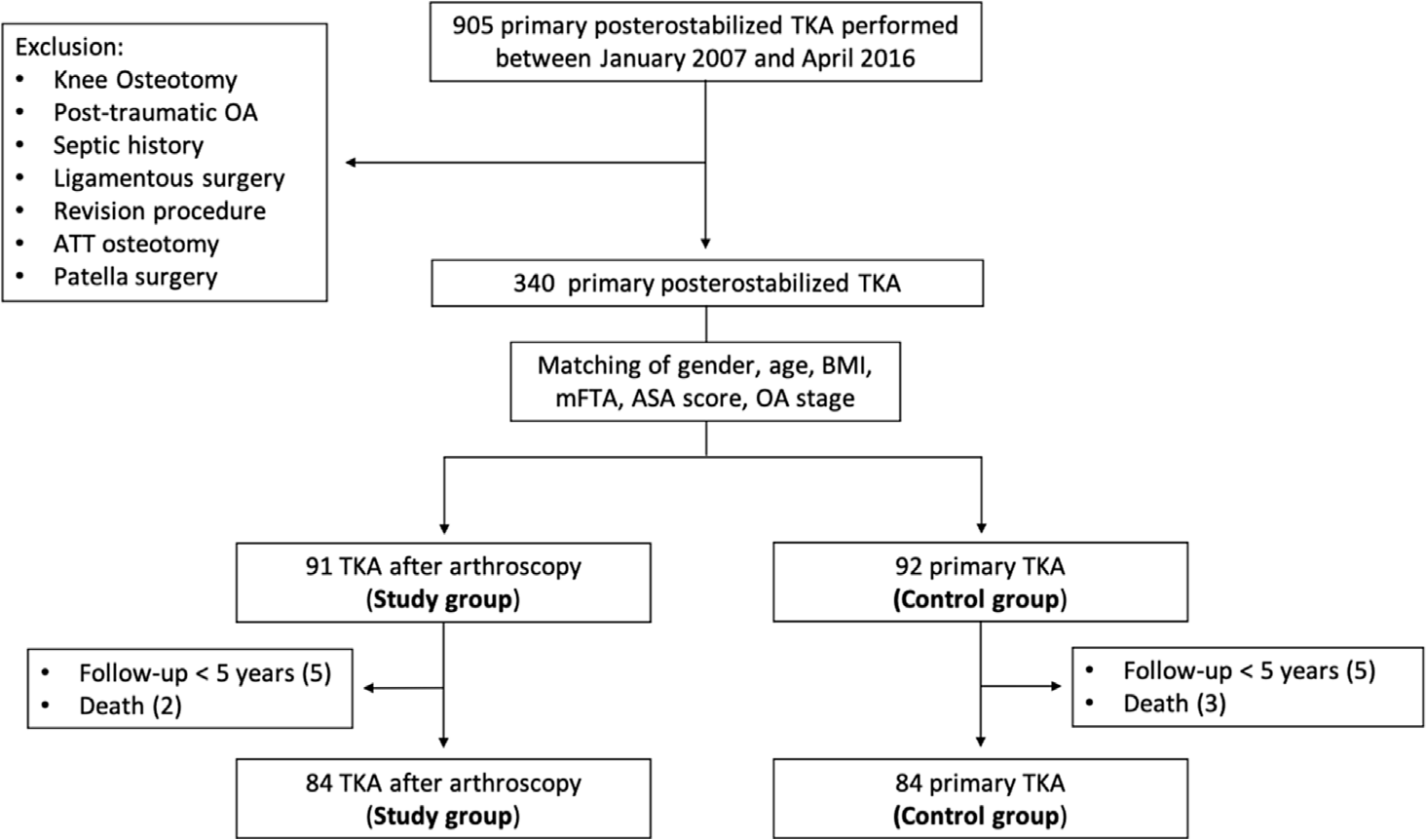
Flowchart depicting the number of the patients (study and control group) enrolled and analyzed in the current study. OA: osteoarthritis; ATT: anterior tibial tubercle; TKA: total knee arthroplasty; BMI: body mass index (kg/m^2^); mFTA: mechanical femorotibial angle; ASA: American Society of Anesthesiologists.

Patients, with a history of KA, due to medial meniscus pathology, such as partial, subtotal, or total medial meniscectomy and/or debridement and meniscal repair, over 18 years old, having undergone TKA due to OA, without any prior open procedures, such as high-tibial or distal femoral osteotomies, internal fixation of proximal tibia fractures or any arthroscopic procedure that involved the cruciate, collateral or the lateral meniscus were included in the study. Furthermore, patients undergoing KA due to isolated chondral lesions were not included. Patients with less than 5 years of follow-up were also excluded (Figure 1).

Initially, 905 patients with a posterior stabilized TKA were found. After excluding the cases with prior surgeries (except KA for medial meniscus), as well as cases of infection and post-traumatic OA, a total of 340 were identified. The groups (study group: with prior KA for medial meniscus pathology, control group: without any prior surgery) were matched for gender, age, body mass index (BMI), mechanical femoro-tibia angle (mFTA), ASA score, OA stage (according to Ahlbäck’s classification [13]). At that point, 91 patients were included in the study and 92 in the control group. After excluding those with less than 5 years of follow-up and those who had passed away the final groups were comprised of 84 patients each.

The mean age of the study group was 68.9 (SD = 7.4) and of the control group 69.1 (SD = 9.1). All patients presented knee osteoarthritis of the medial femorotibial compartment. Arthroscopic procedures included partial, subtotal, or total medial meniscectomy and/or debridement and meniscal repair. All 84 patients of the study group were evaluated clinically and radiographically at a mean follow-up of 8.4 years (±2.1). The control group consisted of patients who did not have any KA and underwent TKA during the same period. A total of 84 patients were identified with a mean follow-up of 8.1 years (±1.8).

All patients were evaluated at the outpatient clinic and underwent thorough clinical examination and radiographic evaluation. Patients were assessed for their symptoms, their range of motion, implant stability, and alignment (objective part) and also for their satisfaction, expectations, and functional activities (subjective component). The IKS [14, 15] was completed. Clinical follow-up was routinely performed at approximately 6 weeks, 1 year, 5 years postoperatively, and every 5 years thereafter. The outcomes included the IKS, all-cause revision, and all re-operation.

## Statistical analysis

Statistical analysis was performed with the online environment EasyMedStat^®^ (http://www.easymedstat.com; Neuilly-SurSeine; France). The distribution of continuous variables was averaged as range and standard deviation. Statistical significance was considered at *p* < 0.05 for all tests.

## Results

### Demographics

After performing a matching process to address demographic inhomogeneity, excluding patients lost to follow-up, and to reduce confounding variable bias, 84 patients with a prior history of KA of the medial meniscus (study group) were included, and 84 patients in the matched control group during this same time frame (Figure 1). The mean follow-up was 8.4 ± 2.1 years (5.1–14.1) and 8.1 ± 1.8 years (5.4–13.8) for the study group and control, respectively.

Table 1 shows the demographic characteristics of the patients. In the study group, 8.3% had stage II OA, followed by 65.5% III and 26.2% IV. In the control group, the patients were classified as stages II, III and IV, in 10.7%, 61.9% and 27.4% respectively. The weight-bearing full-length lower limb (hip–knee–ankle) imaging measured the mFTA angle and showed no significant difference between the two groups. The mean time interval for the study group between KA and TKA was 3.6 years ± 2.4 (range from 0.9 to 17 years).

**Table 1:** Demographic of the cohort.

	Study group (*n* = 84)^a^	Control group (*n* = 84)^a^	*p*-value
Age at surgery (year)	68.9 ± 7.4 [45.9–85]	69.1 ± 9.1 [46.9–87.9]	n.s.
Follow-up (year)	8.4 ± 2.1 [5.1–14.1]	8.1 ± 1.8 [5.4–13.8]	n.s.
Sex (female)	50/84 (59.5%)	52/84 (61.9%)	n.s.
BMI	29.6 ± 5.3 [20.9–44.5]	29.3 ± 4.8 [19.8–41]	n.s.
ASA score			n.s.
– 1	11 (13.1%)	9 (10.7%)	
– 2	46 (54.7%)	43 (51.2%)	
– 3	24 (28.6%)	28 (33.3%)	
– 4	3 (3.6%)	4 (4.8%)	
OA stage (Ahlback)			n.s.
– 1	0	0	
– 2	7 (8.3%)	9 (10.7%)	
– 3	55 (65.5%)	52 (61.9%)	
– 4	22 (26.2%)	23 (27.4%)	
Preoperative mFTA (°)	173.2 ± 4.5 [161–179]	172.7 ± 5.8 [156–179]	n.s.
Preoperative IKS score			
– Knee	53.8 ± 15.1 [15–86]	55.8 ± 12.9 [17–80]	n.s.
– Function	64.7 ± 16.9 [10–100]	60.6 ± 20 [0–90]	n.s.
– Total	118.6 ± 26 [39–159]	114.9 ± 29.4 [0–160]	n.s.

a Data are presented as mean ± standard deviation [minimum–maximum] or number (proportion).

BMI: body mass index (kg/m^2^); ASA: American Society of Anesthesiologists; mFTA: mechanical femorotibial angle; IKS: International Knee Society, n.s.: non-significant.

### IKS score

Preoperatively, the mean IKS knee score did not differ for both groups [53.8 ± 15.1 (range, 15–86) and 55.8 ± 12.9 (range, 17–80), respectively]. Also, the mean IKS functional score showed no difference between the groups [64.7 ± 16.9 (10–100) vs 60.6 ± 20 (0–90)]. In the postoperative follow-up, the mean IKS knee score was 89.4 ± 10.1 [range, 57–100] for the study group and 88.4 ± 12.4 [range, 51–100] for the control group. Similarly, the mean IKS functional score was 79.4 ± 20.3 [range, 20–100] vs 80.7 ± 22.3 [range, 0–100] respectively. The change in IKS knee and functional score from pre-operative to most recent follow-up score was not significantly different between the two groups.

The study group had better post-operative than preoperative IKS knee and functional scores (89.4 ± 10.1, *p* = 0.01 and 79.4 ± 20.3, *p* = 0.03). Additionally, the control group had also a higher post-operative improvement in IKS knee and function scores (88.4 ± 12.4, *p* = 0.02 and 80.7 ± 22.3, *p* = 0.04 respectively). All results are presented in Table 2

**Table 2:** Clinical outcomes.

IKS score type	Study group^a^	Control group^a^	*p*-value
*Preoperative*			
– Knee	53.8 ± 15.1 [15–86]	55.8 ± 12.9 [17–80]	n.s.
– Function	64.7 ± 16.9 [10–100]	60.6 ± 20 [0–90]	n.s.
– Total	118.6 ± 26 [39–159]	114.9 ± 29.4 [0–160]	n.s.
*Postoperative*			
– Knee	89.4 ± 10.1 [57–100]	88.4 ± 12.4 [51–100]	n.s.
– Function	79.4 ± 20.3 [20–100]	80.7 ± 22.3 [0–100]	n.s.
– Total	168.8 ± 25.4 [92–200]	169.1 ± 31.9 [51–200]	n.s.
*Improvement*			
– Knee	34.2 ± 16.7 [−11–80]	33.1 ± 17.2 [−3–100]	n.s.
– Function	14.2 ± 19.3 [−35–60]	19.2 ± 27.2 [−55–100]	n.s.
– Total	48.4 ± 28.4 [−18–124]	52.3 ± 37.4 [−45–200]	n.s.
*p value*			
– Knee	0.01	0.02	
– Function	0.03	0.04	
– Total	0.04	0.03	

IKS: International Knee Score; n.s.: non-significant.

a Data are presented as mean ± standard deviation [minimum–maximum] or number (proportion).

### Complications, revisions, and reoperations

At the mean follow-up of 8.4 ± 2.1 years, we identified 6 postoperative complications, including 3 periprosthetic joint infections and another 3 stiffness cases, in the study group (7.1% cases in this group). A second surgical intervention with a prosthetic component replacement was performed in 3 cases (3.6%). At the mean post-operative follow-up of 8.1 ± 1.8 years 8 complications, including 4 cases of stiffness, 2 of prosthetic joint infection, 1 of patellar tendon rupture and 1 of patella baja, were identified in the control group (9.5% of cases). Three cases underwent revision surgery (3.6%). Regarding the risk of revision and the risk of reoperation, we observed that there was no statistical difference between the study and control group (Table 3).

**Table 3:** Complications and revisions outcomes.

	Study group (*n* = 84)^a^	Control group (*n* = 84)^a^	*p*-value
Postoperative complications	6 (7.1%)	8 (9.5%)	n.s.
Revision implant	3 (3.6%)	3 (3.6%)	n.s.

a Data are presented as mean ± standard deviation [minimum–maximum] or number (proportion).

## Discussion

Limited information exists on the results of initial total knee arthroplasty (TKA) in individuals who previously had undergone knee arthroscopy for medial meniscus, including partial, subtotal, or complete removal, as well as repair. While certain studies propose that prior knee arthroscopy might heighten the risk of complications during TKA, contrasting research findings suggest no adverse effects on TKA outcomes [9, 10]. The objective of this study was to compare the outcomes of TKA in two groups: one comprising patients who had undergone prior KA for medial meniscus pathologies (study group) and the other consisting of patients undergoing TKA without prior KA (control group). The evaluation included examining the IKS, complications, and revision rates. The main findings of the study are that patients with previous KA due to medial meniscus pathology and subsequent TKA have similar functional outcomes, complications, and revision rates compared to those without previous KA, with more than 8 years of follow-up.

This study has some limitations. Firstly, it is of a retrospective nature with relatively small samples originating from a single center. Furthermore, this study did not evaluate the optimum time interval between TKA and KA and did not sub-analyze the impact of different procedures, such as meniscectomy compared to meniscus repair, on the outcome of TKA. This information was not available due to the retrospective nature of the study. Nevertheless, it includes more than 5 years of follow-up and a matched comparison between the two groups adding valuable insights to this debatable issue in the literature.

It has been reported that previous open knee interventions like high tibial osteotomies are correlated with inferior functional and clinical outcomes in patients undergoing TKA when compared with those of primary TKA without previous procedures [16–18]. Moreover, TKA patients with prior anterior cruciate ligament (ACL) reconstruction have also exhibited inferior outcomes. In particular, a recent retrospective study by Watters et al. of 122 patients undergoing TKA with a prior history of anterior cruciate ligament (ACL) reconstruction found a higher risk of early re-operation [19].

On the other hand, there is only data on the results of TKA following non-ligamentous KA, such as debridement and partial meniscectomy [11, 12, 20, 21]. KA is considered minimally invasive; however, it could lead to post-operative complications such as further cartilaginous damage, arthrofibrosis, adhesions, infections, or cardiovascular problems [22]. In this retrospective cohort study, we suggest that prior KA for medial meniscus pathology should not be considered a risk factor for revision surgery to subsequent TKA.

In the present work, patients with or without previous KA of medial meniscus and subsequent TKA improved the IKS score following TKA, but they did not reveal significant differences in functional outcomes at the final follow-up. Similar to the present study, Viste et al. [12] retrospectively reviewed 480 TKAs with or without a prior non-ligamentous history of KA, including debridement, loose body removal, partial meniscectomy, and chondroplasty. They reported similar Knee Society Score (KSS) among the groups at the most recent follow-up. Piedade et al. performed a retrospective cohort study of 60 patients undergoing TKA after knee arthroscopic debridement compared to the control group [23]. The authors reported no difference in the pre- and post-operative IKS knee score for both groups.

On the other hand, some studies have shown the negative impact of previous KA on the functional outcomes of a primary TKA. Hu et al. [24] performed a retrospective matched cohort study of 68 patients (70 knees) who underwent TKA following KA for debridement with a mean follow-up period of 3.2 years. They emphasized that prior KA is associated with reduced functional outcomes or increased risks of revision and complications following TKA. Moreover, a retrospective propensity score matching-based control study reviewed 92 primary TKAs with a history of prior KA due to meniscus tears, chondromalacia or ACL injury from 2013 to 2017 showing that prior KA is associated with worse clinical outcomes in patients with prior KA, especially in males and those with prior KA for ACL injury [20]. Barton et al. [25], in a retrospective review of 186 patients who underwent TKA and subdivided to TKA within six months and within six to twelve months of KA, concluded that there is a negative impact on functional outcomes (Oxford Knee Score) of the subsequent TKA which seems to be time dependent. They also state that TKA should not be routinely performed within six months of KA.

Several studies have also suggested that previous KA, encompassing medial et lateral meniscectomies, debridement, or washouts, loose body removals and ACL debridement combined with meniscectomy, resulted in higher rates of postoperative complications, revisions and periprosthetic joint infection of the subsequent TKA [11, 25, 26]. The present comparative study revealed that the complication rate was 7.1% and 9.5% (*p* > 0.05) for the study (patients with prior KA) and the control group (patients without history of KA or open procedure), respectively. Additionally, revisions were found to be equivalent between the two groups at a rate of 3.6% for a mean follow-up of more than 8 years. Lubowitz et al. [27] reported similar complication rates, ranging from 7.2% to 8.1% for TKA without and with ipsilateral KA debridement. In a more recent study, Gu et al. [11] showed an association between prior KA for debridement, medial tear of meniscus or chondromalacia, and complications after TKA. They also found that revision rates were significantly associated with prior KA at 2-year follow-up.

It is of paramount importance to examine the time-dependent impact of KA upon outcomes ensuing TKA. In this investigation, the mean time interval between KA and TKA was 3.6 years ± 2.4 (range from 0.9 to 17 years). The optimal time to perform TKA following KA is controversial. A study by Werner et al. [28] concluded that the incidence of infection, stiffness, and venous thromboembolism was higher in patients who underwent TKA within six months after knee arthroscopy compared with the patients in the control group. Similarly, Barton et al. [25] yielded that the interval period was a crucial factor for favorable results. They demonstrated that the functional outcomes were significantly reduced when TKA was performed within 6 months. In contrast, there was no significant difference when the patients underwent TKA between six months and 12 months after KA. On the contrary, Piedade et al. [23] did not reveal a direct correlation between KA and TKA interval to postoperative complications or failures with a mean interval of 53 months. According to Viste et al. [9] time interval between previous non-ACL arthroscopic procedures and TKA did not reveal any increased risk of infection or other complications and revision procedures.

This work primarily investigates the impact of prior KA for medial meniscus pathology on TKA, revealing that it may not pose a significant risk factor for revision surgery. However, it is of note that other types of KA, such as those involving the cruciate ligaments, are both menisci or other entities. Several studies have demonstrated that TKA in a previous ACL reconstruction is associated with a notable increased risk of complications, extended duration of surgery procedures, and a higher need for revision components [17, 27]. A recent retrospective study showed that patients with previous medial and lateral meniscectomy might experience inferior short-term functional outcomes following TKA [28]. Conversely, the outcomes of the varied nature of arthroscopic knee interventions, such as debridement for OA, washouts, or loose body removals seem to yield conflicting results in the existing literature. Some authors report no discernible differences in outcomes [10, 19] while others suggest a decline in patients’ outcomes afterwards a TKA [9, 23]. While the present study focuses on medial meniscus pathology, further comprehensive investigations are required to elucidate whether the observed trends hold true for a broader spectrum of knee arthroscopic meniscal procedures, ensuring a more comprehensive understanding of their impact on TKA.

## Conclusion

The present study seems to demonstrate that previous KA on the medial meniscus, including partial, subtotal, or total medial meniscectomy and/or debridement and meniscal repair, does not have a negative impact on a future TKA, in terms of functional outcomes, complications, and revision rates. Many patients undergoing TKA have had KA in the past and more data and large prospective comparative studies are needed to determine the possible impact that a previous KA could have on TKA.

## Conflict of interest

The authors declare that they have no relevant financial or non-financial interests related to this work.

VG, AS, AV, CK, CB: declare that they have no conflict of interest.

ES: Institutional research support from Corin.

SL: Consultant for Stryker, Smith and Nephew, Heraeus, Depuy Synthes. Institutional research support to Lepine and Amplitude. Editorial Board for Journal of Bone and Joint Surgery (Am).

## Funding

This research did not receive any specific funding.

## Ethical approval

Ethical approval was not required.

All procedures were performed in accordance with the ethical standards of the institutional and/or national research committee, the 1964 Helsinki Declaration and its later amendments, or comparable ethical standards. Data collection and analysis were carried out in accordance with MR004 Reference Methodology from the Commission Nationale de l’Informatique et des Libertés (Ref. 2226075) obtained the 19 April 2022.

## Informed consent

Written informed consent was obtained from all patients and/or families.

## Authors contributions

V. Giovanoulis: Conceptualization, methodology, data curation, writing an original draft. A. Schmidt: Data curation, writing, reviewing, and editing. A. Vasiliadis: Conceptualization, data curation, writing, reviewing. C. Koutserimpas: conceptualization, writing, reviewing, and editing. C. Batailler: writing, reviewing, and editing. S. Lustig: conceptualization, supervision, validation, writing, reviewing, and editing. E. Servien: conceptualization, methodology, data curation, supervision, validation, writing, reviewing, and editing.
